# Efficacy of minimally invasive nonthermal laser-induced optical breakdown technology for skin rejuvenation

**DOI:** 10.1007/s10103-012-1179-z

**Published:** 2012-08-14

**Authors:** Louis Habbema, Rieko Verhagen, Robbert Van Hal, Yan Liu, Babu Varghese

**Affiliations:** 1 Department of Dermatology, Medisch Centrum ’t Gooi, Bussum, The Netherlands; 2 Care and Health Applications Group, Philips Research Europe, Eindhoven, The Netherlands

**Keywords:** Skin rejuvenation, Collagen, Wrinkle reduction, Laser-induced optical breakdown

## Abstract

We demonstrate the efficacy of a novel minimally invasive nonthermal skin rejuvenation technique for wrinkle and fine-line reduction based on laser-induced optical breakdown. The optical breakdown caused by tightly focused near-infrared laser pulses creates a grid of intradermal lesions without affecting the epidermis, leading to skin rejuvenation. The pilot in vivo efficacy test performed on five subjects successfully demonstrates wrinkle and fine‐line reduction, and improvement of other skin features without pain or any other unpleasant sensations or any social downtime associated with the treatment. The efficacy is evaluated objectively and subjectively by assessing the improvement of wrinkles and/or fine lines or skin texture after the treatment. The treatment is safe without side effects or social downtime, and all test subjects reported that the treatment is “perceptible but not painful.” Four out of the five subjects who participated in this pilot study were assessed to have “minor” to “significant” improvements of wrinkles and fine lines by the professional panels. The results of this clinical study are expected to bring a paradigm shift in the present laser- and light-based skin rejuvenation methods by introducing a safe treatment procedure without damaging the epidermis, with no or little social downtime and with an efficacy that might be comparable to ablative techniques.

## Introduction

The present laser- and light-based ablative, nonablative and fractional skin rejuvenation techniques rely on selective photothermolysis based on linear absorption of optical energy by the skin’s constituents [1–6]. The prolonged recovery time and significant risk profile associated with the highly effective ablative techniques prompted the development of nonablative and fractional methods [3, 4]. Nonablative fractional photothermolysis creates thermal damage in the dermis without causing significant epidermal removal or injury. Even though these methods are becoming more popular due to the lower risks, the clinical results showed limited efficacy [5, 6]. Furthermore, with multiple passes, the ablative damage accumulates, which increases the thermal damage and healing time. In spite of technological improvements in this field throughout the years, no revolutionary approach has been introduced that is capable of accurately defining the balance between efficacy, safety, social downtime, and pain perception.

**Fig. 1:**
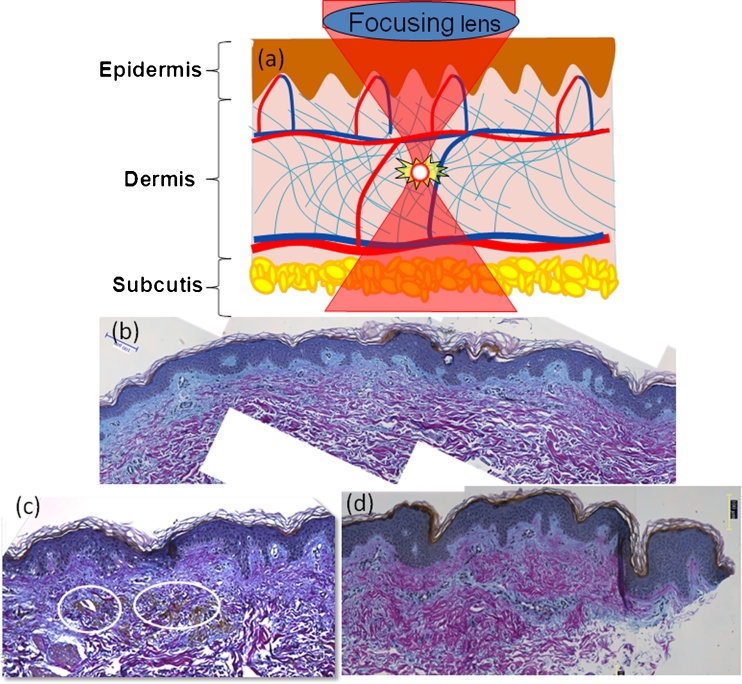
Schematic representation of lesion creation in dermis using laser-induced optical breakdown (**a**). Skin specimen taken 30 min after treatment, stained with Herovici staining. Control skin specimen stained with Herovici staining (**b**). *Circled* are microlesions visualized by the staining technique (**c**). Skin specimen taken at 30 days after treatment was stained with Herovici staining. The mature collagen (collagen I) stained in *red* whereas the young collagen (collagen III) stained in *blue*

Recently, we have introduced a novel minimally invasive skin rejuvenation modality, stimulating selective dermal collagen production and remodeling, without disrupting the epidermal surface, and with little or no healing time and reduced patient discomfort [7]. The method introduced here is fundamentally different from the previously reported ablative, nonablative, and fractional laser-based skin rejuvenation methods based on selective photothermolysis. In this method, the optical breakdown caused by tightly focused near-infrared laser pulses creates a grid of intradermal lesions that lead to skin rejuvenation without affecting the epidermis (Fig. 1). To introduce this novel technology, which could potentially create a paradigm shift in the present laser-based skin rejuvenation arena, we have used a phased approach because it is not desirable to perform an extended clinical trial on the facial skin. In the first phase, we have demonstrated creation of intradermal lesion on ex vivo skin, leaving epidermis intact. In the next phase, we showed the evidence of microscopic lesion creation and new collagen formation at the sites of the optical breakdown in the days following the in vivo treatment performed on five test subjects on buttock skin. The goal of the present clinical study is to investigate the efficacy of this novel laser-induced optical breakdown technology for wrinkle reduction based on the clinical study performed on five subjects. The primary clinical end point of this study is the improvement of wrinkles and/or fine lines or skin texture posttreatment, as determined objectively by the blinded assessment performed by trained observers and a team of dermatologists and subjectively by the principal investigator and subjects. The secondary end points of this study are the assessment of the severity of the side effects by the principal investigator and of user comfort and discomfort by the subjects.

## Materials and methods

The clinical study was performed with an in-house prototype device [7]. The device has obtained a declaration of European Conformity by Philips Electronics N.V. and was reviewed and approved by the Medical Ethics Committee (Medisch Ethische Toetsings Commissie). Informed consent was obtained prior to the subject’s participation. The prototype device consists of a base station, an articulated arm, a treatment hand piece, and a computer. The base station houses an optical system, a cooling system, and electronics. The optical system comprises a pulsed laser source, beam shaping optics, and mirrors to guide the laser beam to the handpiece via the articulated arm. The laser source is a flash lamp pumped SLM TEM00 Nd/YAG laser which delivers subnanosecond light pulses of 1,064 nm with pulse energies in excess of 0.15 mJ at focus level inside the skin, which is sufficient to cause optical breakdown. The handpiece consists of a focusing system that focuses the laser beam to a focal spot (Ф < 10 μm) within the skin, sufficient to cause optical breakdown, resulting in a cavitation bubble in the skin. The cavitation bubble increases the area of damage, and the lesion size ultimately reaches 0.1 to 0.2 mm in diameter [8–10]. During treatment, an optical matching liquid is applied to effectively couple the light to the skin. The optical scanner integrated inside the handpiece is able to treat a selected area of the skin (19 × 19 mm^2^) at a scanning speed of up to 5 mm/s with a focus depth which is adjustable in the range of 100 to 750 μm below the surface of the skin. The scan pattern is controlled by an algorithm implemented in a computer. The pitch of the grid of laser lesions inside the skin can be adjusted in the range of a few micrometers up to several millimeters. A glass plate is used to protect the optical elements from direct contact with the treated skin surface and to define the depth of treatment in the skin.

### Study design and procedure

This study was conducted at Medisch Centrum ‘t Gooi in Bussum, The Netherlands. Inclusion criteria required subjects to be aged between 35 and 60 years, to have mild to moderate wrinkles or the presence of fine lines, and have Fitzpatrick skin types of I–IV. The study comprised a full treatment performed in five sessions and three follow-up visits (at intervals of 1 week, 1 month, and 3 months) after the completion of the final session for evaluation. The principal investigator selected the treatment zones on the subject’s facial area (e.g., perioral, periorbital, and cheek) where wrinkles or fine lines were present. The size of a single treatment area was 8 × 8 mm^2^. The coverage value can be varied from 2.5 to 20 %. Before the treatment, the principal investigator treated two small areas (4 × 4 mm^2^) on the cheek and near the ear with a predetermined coverage value (e.g., 10 %) to assess whether the perception and side effects (if any) were acceptable to the subject and the principal investigator. Subjects 1 and 2 were treated on periorbital area; subjects 3 and 4 were treated on their cheek area; and subject 5 was treated on perioral area.

The sensations perceived by the test subjects during the treatment were assessed by means of a questionnaire for evaluating the subjective experience of the treatment. The type and severity of postsession skin responses, such as pigmentary changes, pin bleeding, and any other side effects were noted and documented for evaluating whether the severity of the observed side effects were accepted by the test subjects. Furthermore, the dermatologist assessed the skin responses during and after the treatment.

The objective assessment was based on the clinical photographs taken before and after the treatment by a panel consisting of three independent “blinded” dermatologists, not involved in this clinical trial and seven experts trained in the photo assessment for cosmetic-related studies. All independent observers assessed the wrinkle grade of photos based on a 10-point Fitzpatrick wrinkle grade [11] (0, no lines; 1–3, fine lines; 4–6, fine to moderate-depth wrinkles, moderate number of lines; and 7–9, moderate to deep wrinkles, numerous lines with or without redundant skin folds). For the subjective analyses, the patient satisfaction index was recorded on a 5-point scale (0, no change; 1, minor-mild improvement; 2, mild-moderate improvement; 3, significant improvement; and 4, almost complete clearing effect).

## Results and discussion

### Objective assessment: improvement as rated by a panel of dermatologists

**Fig. 2:**
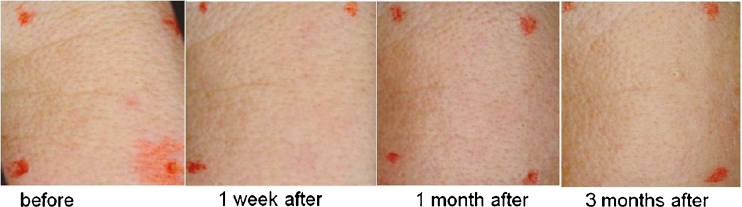
Photos of subject 1 before the treatment and 1 week, 1 month, and 3 months posttreatment

**Fig. 3:**
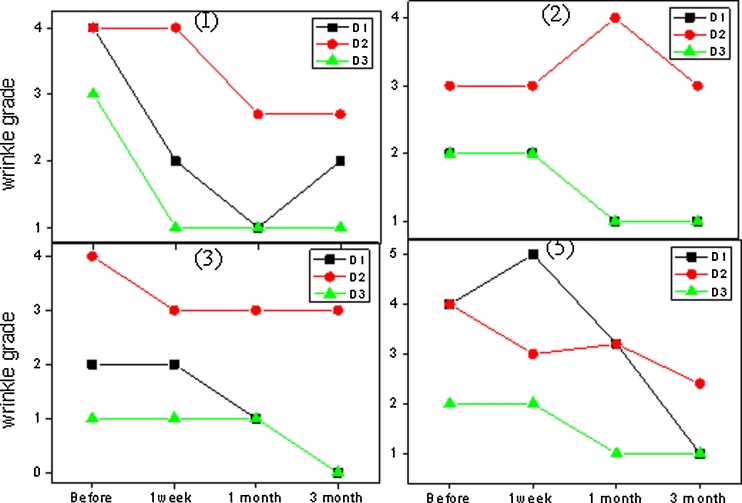
Fitzpatrick wrinkle grade (*0*, no lines; *1–3*, fine lines; *4–6*, fine to moderate-depth wrinkles, moderate number of lines; *7–9*, moderate to deep wrinkles, numerous lines with or without redundant skin folds) for subjects 1, 2, 3, and 5, assessed by three dermatologists (D1, D2, and D3) before, 1 week, 1 month, and 3 months after treatment

**Fig. 4:**
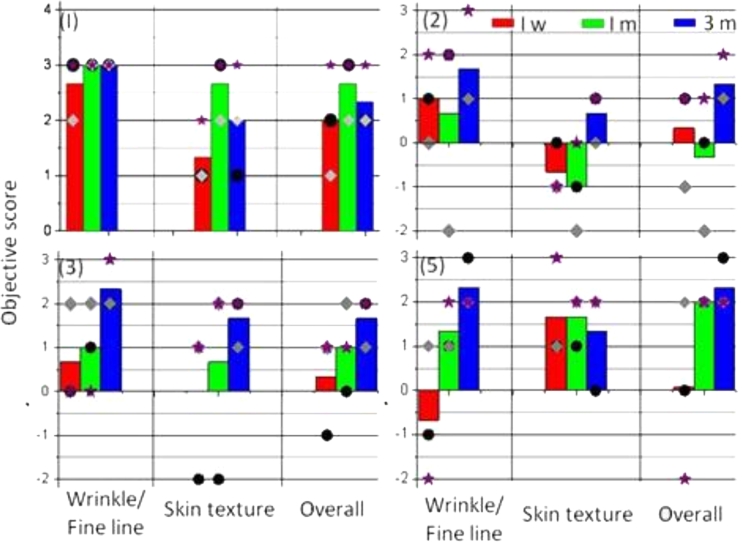
Mean improvements of skin features for subjects 1, 2, 3, and 5 assessed by the dermatologist team at different timelines after the completion of treatment: 1 week (*red bar*), 1 month (*green bar*), and 3 months (*blue bar*). Individual score of improvement from each dermatologist is differentiated by three symbols: dermatologist 1 (*circles*), dermatologist 2 (*diamonds*), and dermatologist 3 (*stars*), respectively

Fitzpatrick wrinkle grade and mean improvements of each given skin feature assessed by three dermatologists based on the clinical photographs (Fig. 2) before treatment and at 1 week, 1 month, and 3 months intervals after treatment are shown in Figs. 3 and 4. The mean improvements rated by the three dermatologists are consistent with each other. In general, all test subjects except subject 4 were assessed by dermatologists as having reduction of Fitzpatrick wrinkle grade after treatment. The overall appearance of skin was also improved, ranging from “moderate” to “significant.” Subject 4 started with wrinkle grade of 0, and nearly no improvements were observed by the dermatologists’ team, and the results are therefore not shown in Figs. 3 and 4.

### Objective assessment: improvement as rated by trained observers

**Fig. 5:**
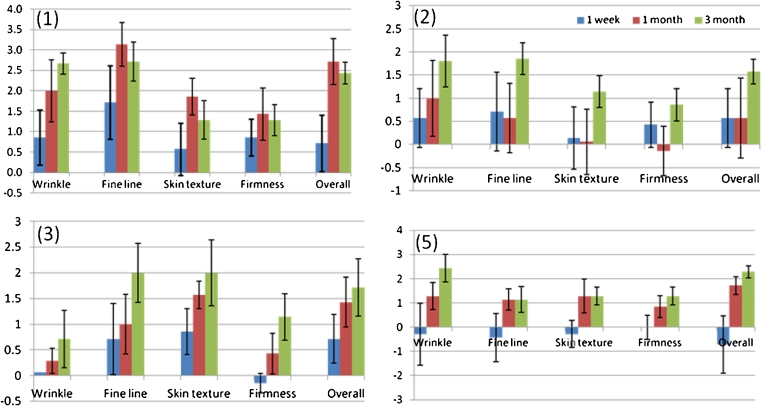
The mean improvements of skin features assessed by the independent expert panel

The mean improvements of skin features observed for all subjects assessed by the independent expert panel are shown in Fig. 5. For all subjects except for subject 4, the scores improved from 1 week to 3 months. For subject 4, nearly no wrinkle is visible from the close-up photos. As a consequence, no improvement of wrinkle or fine line was observed; nor for other skin features. This correlated with the scores provided by the dermatologists. Subject 5 received the treatments on perioral area where a relatively deeper wrinkle was present. No obvious wrinkle reduction was observed 1 week after the treatment. The independent observers’ scores were below 0, which implied that most of the independent observers picked up a wrong picture as a posttreatment picture. However, clear improvement of wrinkle and fine line was seen 1 and 3 months after treatment.

For subject 2, additional skin feature improvement, e.g., dyschromia, is noted by three of seven independent observers. This implies that the skin looks more uniform after the treatments. For subject 3, the main improvement was observed for fine line, skin texture, and overall appearance. Scar reduction was also observed for subject 3 by the majority of all independent observers. The improvement was assessed as “moderate” to “significant.” The pictures taken at 3 months after treatment received the highest score (moderate to very significant) of all listed skin features as assessed by all observers. Furthermore, after the treatment, skin looked more even.

### Subjective assessment: improvement as rated by the subjects and principal investigator

The scores perceived by the subjects themselves and the principal investigator (PI) are shown in Table 1. Two subjects (subjects 1 and 2) who were treated on their periorbital area perceived more improvements than the other three subjects who were treated on the cheek (subjects 3 and 4) and perioral area (subject 5), respectively. The improvements assessed by the PI for subjects 3, 4, and 5 are also not significant and therefore scores are not shown in Table 1. The perceived results 3 months after the completion of the treatment are in general lower than those perceived 1 week and 1 month posttreatment. The observation that the periorbital area appeared to be more responsive than the perioral area support the results of a previous nonablative laser skin resurfacing study performed using 1,540 nm erbium glass laser [12]. Skin at the periorbital area is relatively thin, implying increased dermal wounding, and new collagen formation compared with other facial areas, which made the subjective assessment much easier than those being treated at other facial areas. Test subjects perceived significant improvements based on the photographs taken before and after the treatment.

**Table. 1:** Scores (0, no change; 1, minor-mild improvement; 2, mild-moderate improvement; 3, significant improvement; and 4, almost complete clearing) perceived by subjects 1 and 2 and principal investigator (PI) 1 week, 1 month, and 3 months respectively after the treatment

Subjective assessment by	ID No.	Wrinkle reduction			Fine line			Skin texture			Firmness			Overall improvement		
		1 week	1 month	3 months	1 week	1 month	3 months	1 week	1 month	3 months	1 week	1 month	3 months	1 week	1 month	3 months
Subject	1	1	0	0	2	1	1	3	2	1	1	1	1	2	2	1
PI	1	1	0	0	1	0	2	3	0	3	0	0	0	2	0	2
Subject	2	3	4	3	4	4	4	2	3	2	2	2	2	3	4	2
PI	2	4	1	2	4	2	3	3	3	2	2	0	1	3	2	2

### Side effect profile: skin responses and treatment sensations

In all treatment sessions, minor to mild erythema occurred in all subjects immediately posttreatment and was resolved a maximum of 20 min after the treatment. Additional posttreatment response, i.e, minor edema observed in one subject 10 min after the treatment was resolved within 20 min. Petechiae (tiny intradermal bleeding) occurred in one subject during one treatment because of the incorrect placement of the treatment head. After the replacement of the treatment handpiece, no more petechiae were seen. No hyper- or hypo-pigmentation was observed after the resolving of the petechiae. No other un-anticipated skin reactions were observed during and after the treatment sessions. Sensations perceived during the treatment ranged from “not perceptible” to “perceptible.” In all cases, no pain sensation was reported.

The common adverse effects of ablative laser treatment include prolonged postoperative erythema, edema, acneiform eruptions, milia formation, dyspigmentation, hypertrophic scar formation, and delayed wound healing [1]. For these reasons, the recent trend in laser technology is to develop alternate and less invasive methods of skin rejuvenation. In fractional resurfacing or fractional photothermolysis, microthermal injury zones of approximately 70–150 μm in width and 400–700 μm depth are created, leaving intervening areas of normal skin intact. Today, we have some less invasive treatment options commercially available, such as Fractional 1540, before considering CO_2_ laser resurfacing. The clinical improvements obtained with this novel technology are comparable to nonablative Er/YAG lasers. Here, the handpiece delivers light in an array of narrow, focused high-precision microbeams to create narrow, deep columns of heat or tissue coagulation in the epidermis and upper dermis while keeping the stratum corneum in place. The heated tissue within these treated columns then initiate a natural healing process that forms a new healthy tissue. Together, these features make this procedure safe and predictable while reducing discomfort and the risk of scarring and pigmentation problems. If the laser is applied with multiple passes for significant improvement in wrinkle reduction, the ablative damage accumulates, which increases thermal damage and healing time [13]. In our method, highly confined energy leads to a localized mechanical effect in the form of a microexplosion [7] in the dermis and thus a larger coverage area can be used. This allows for a larger proportion of the dermal collagen to be regenerated, without the risk of severe side effects. Furthermore, the treatment is pain free, which is a unique desired feature of this method. With higher energy, coverage settings, and multiple depths, clinical improvements can also be made significant. But, we would expect the subjective sensation will probably will tend to change from perceptible but not painful to perceptible and slightly painful. In general, the coverage areas of these zones are in the range of 5 to 20 % of the skin surface area per treatment session. Furthermore, because the new technique generates lesions at the dermal level where the formation of new collagen will occur, we consider that the efficacy of the new technique will be comparable to conventional fractional ablative techniques. The level of safety is the same as with nonablative techniques because it generates microlesions in the dermis while the epidermis is unaffected. This technique enables a breakthrough skin rejuvenation method by introducing a safe treatment procedure without damaging the epidermis and with no or little social downtime. The efficacy and sensation results demonstrated here are based on five subjects and therefore it is premature to quantify the efficacy and conclude that there are no side effects. In the present phase, we are performing an extended clinical study to obtain statistically significant and clinically relevant outcomes. These results of this study will be reported separately in the near future.

## Conclusions

In this clinical study, we have successfully demonstrated the efficacy of a novel minimally invasive nonthermal skin rejuvenation technique for wrinkle and fine-line reduction based on laser-induced optical breakdown. This pilot in vivo efficacy test performed on five subjects successfully demonstrates wrinkle, fine-line reduction, and improvement of other skin features without pain or any other unpleasant sensations or any social downtime associated with the treatment. The perception of the treatment was found to be acceptable for the majority of the test panel, without the use of topical local or systemic anesthesia. The sensations perceived ranged from “not perceptible” to “perceptible.” The results of this clinical study are expected to bring a paradigm shift in the present laser- and light-based skin rejuvenation methods by introducing a safe treatment procedure without damaging the epidermis, with no or little social downtime and with an efficacy that might be comparable to ablative techniques.

## References

[1] Bernstein LJ, Kauvar AN, Grossman MC, Geronemus RG (1997). The short- and long-term side effects of carbon dioxide laser resurfacing. Dermatol Surg.

[2] Papadavid E, Katsambas A (2003). Lasers for facial rejuvenation: a review. Int J Derma.

[3] Hohenleutner S, Koellner K, Lorenz S, Hohenleutner S (2005). Results of nonablative wrinkle reduction with a 1450 nm diode laser: difficulties in the assessment of subtle changes. Lasers Surg Med.

[4] Ross EV, Sajben FP, Hsia J, Barnette D, Miller CH, McKinlay JR (2000). Nonablative skin remodeling: selective dermal heating with a mid-infrared laser and contact cooling combination. Lasers Surg Med.

[5] Grema H, Greve B, Raulin C (2003). Facial rhytides—subsurfacing or resurfacine? A review. Lasers Surg Med.

[6] Kan M, Choi B, Chess S, Kelly KM, McCullough J, Nelson JS (2004). Optical clearing of *in-vivo* human skin: implications for light-based diagnostic imaging and therapeutics. Lasers Surg Med.

[7] Habbema L, Verhagen R, Hal R, Liu Y, Varghese B, Minimally invasive non-thermal laser technology using laser-induced optical breakdown for skin rejuvenation. Journal of Biophotonics. doi: 10.1002/jbio.201100083. 10.1002/jbio.201100083 PMC3494308 22045580

[8] Kennedy PK, Hammer DX, Rockwell BA (1997). Laser-induced breakdown in aqueous media. Prog Quantum Electron.

[9] Vogel A, Lauterborn W (1988). Acoustic transient generation by laser–produced cavitation bubbles near solid boundaries. J Acoust Soc Am.

[10] Vogel A, Busch S, Parlitz U (1996). Shock wave emission and cavitation bubble generation by picosecond and nanosecond optical breakdown in water. J Acoust Soc Am.

[11] Fitzpatrick RE, Goldman MP, Satur NM, Tope WD (1996). Pulsed carbon dioxide laser resurfacing of photo-aged facial skin. Arch Dermatol Apr.

[12] Lupton JR, Williams CM, Alster TS (2002). Nonablative laser resurfacing using a 1540 nm Ebium Glass laser: a clinical and histologic analysis. Dermatol Surg.

[13] Clementoni MT, Gilardino P, Muti GF, Beretta D, Schianchi R (2007). Non-sequential fractional ultrapulsed CO_2_ resurfacing of photoaged facial skin: preliminary clinical report. J Cosmet Laser Ther.

